# Towards a core-set of mobility measures in ageing research: The need to define mobility and its constructs

**DOI:** 10.1186/s12877-023-03859-5

**Published:** 2023-04-06

**Authors:** Esmee M. Reijnierse, Sven J.G. Geelen, Marike van der Schaaf, Bart Visser, Rob C.I. Wüst, Mirjam Pijnappels, Carel G. M. Meskers

**Affiliations:** 1 Department of Rehabilitation Medicine, Amsterdam UMC location Vrije Universiteit Amsterdam, De Boelelaan 1117, Amsterdam, The Netherlands; 2 Amsterdam Movement Sciences, Ageing & Vitality, Amsterdam, The Netherlands; 3 Centre of Expertise Urban Vitality, Faculty of Sports and Nutrition, Amsterdam University of Applied Sciences, Dokter Meurerlaan 8, Amsterdam, 1067 SM The Netherlands; 4 Department of Rehabilitation Medicine, Amsterdam Movement Sciences, Amsterdam UMC, University of Amsterdam, Meibergdreef 9, Amsterdam, 1105 AZ The Netherlands; 5 Centre of Expertise Urban Vitality, Faculty of Health, Amsterdam Movement Sciences, Amsterdam University of Applied Sciences, Tafelbergweg 51, Amsterdam, 1105 BD The Netherlands; 6 Department of Human Movement Sciences, Faculty of Behavioural and Movement Sciences, Amsterdam Movement Sciences, Vrije Universiteit Amsterdam, Van der Boechorststraat 7, Amsterdam, 1081 BT The Netherlands

**Keywords:** Mobility limitation, Physical capacity, Physical performance

## Abstract

**Background:**

Mobility is a key determinant and outcome of healthy ageing but its definition, conceptual framework and underlying constructs within the physical domain may need clarification for data comparison and sharing in ageing research. This study aimed to (1) review definitions and conceptual frameworks of mobility, (2) explore agreement on the definition of mobility, conceptual frameworks, constructs and measures of mobility, and (3) define, classify and identify constructs.

**Methods:**

A three-step approach was adopted: a literature review and two rounds of expert questionnaires (n = 64, n = 31, respectively). Agreement on statements was assessed using a five-point Likert scale; the answer options ‘strongly agree’ or ‘agree’ were combined. The percentage of respondents was subsequently used to classify agreements for each statement as: strong (≥ 80%), moderate (≥ 70% and < 80%) and low (< 70%).

**Results:**

A variety of definitions of mobility, conceptual frameworks and constructs were found in the literature and among respondents. Strong agreement was found on defining mobility as the ability to move, including the use of assistive devices. Multiple constructs and measures were identified, but low agreements and variability were found on definitions, classifications and identification of constructs. Strong agreements were found on defining physical capacity (what a person is maximally capable of, ‘can do’) and performance (what a person actually does in their daily life, ‘do’) as key constructs of mobility.

**Conclusion:**

Agreements on definitions of mobility, physical capacity and performance were found, but constructs of mobility need to be further identified, defined and classified appropriately. Clear terminology and definitions are essential to facilitate communication and interpretation in operationalising the physical domain of mobility as a prerequisite for standardisation of mobility measures.

**Supplementary Information:**

The online version contains supplementary material available at 10.1186/s12877-023-03859-5.

## Background

Standardization of measures in ageing research for data comparison and sharing requires uniform definitions and nomenclature. A key determinant and outcome of healthy ageing is mobility [1]. Impaired mobility is reported to be prevalent in 46% of older adults [2] and associated with negative health outcomes such as dependency in activities of daily living (ADL) [3], institutionalization [4], poor quality of life [5] and mortality [6]. There are multiple risk factors for impaired mobility, such as walking impairment [1], injuries [1], falls [7], cognitive impairment [8], comorbidity and psychosocial factors [9]. Nevertheless, despite its apparent clinical importance, mobility is not uniformly defined. Mobility commonly refers to the movement of oneself or is referred to in the context of travel and commuting [10–12]. Furthermore, mobility can be conceptualised using multiple interrelations within conceptual frameworks [13] and encompasses various poorly defined underlying constructs and measures [14].

The World Health Organization (WHO) International Classification of Functioning, Disability and Health (ICF) defines mobility in a general way as “movement by changing body position or location or by transferring from one place to another” [15]. This conceptual framework includes components of body functions and structures, activities, participation, environmental factors and personal factors [15]. Other frameworks encompass specific mobility domains, i.e. financial, psychosocial, environmental, physical, cognitive, and gender, cultural and biographical influences [11], or focus on risk factors of mobility limitation [9]. However, none of these frameworks includes a comprehensive view of the physical domain; a domain of high relevance in ageing research and clinical practice. Moreover, within these frameworks, mobility encompasses multiple underlying and poorly defined constructs within the physical domain, such as physical performance and physical capacity, which are often used interchangeably while having different meanings [16]. Furthermore, mobility constructs and measures may depend on how they are assessed, i.e. a standardized environment versus daily life [7, 17] and this may also depend on the research question or clinical problem in which they are operationalized [18].

Clarity on terminology, definitions, conceptual frameworks and constructs is required to develop standardized sets of mobility measures for determinants and outcomes in ageing research, needed to facilitate data comparison and sharing following the FAIR (Findable, Accessible, Interoperable, Reusable) data principles [19]. Therefore, we aimed, based on existing literature and expert opinions, (1) to review definitions and conceptual frameworks on mobility, (2) to explore agreement on the definition of mobility, conceptual framework, constructs and measures of mobility within the physical domain, and (3) to further define, classify and identify the relevance of constructs within the physical domain as a first step to reach a formal consensus.

## Methods

### Study design

A three-step approach was adopted: a literature review was performed and results were discussed among the authors, encompassing experts in ageing research, human movement sciences, muscle physiology, physical therapy and rehabilitation. Results of the literature review evolved in drafting and executing two rounds of expert questionnaires (**Additional** Tables 1 and 2) to explore the agreement on the definition of mobility, its conceptual framework and constructs, and measures of mobility (questionnaire 1) and to further define, classify and identify the relevance of constructs of mobility (questionnaire 2) within the physical domain of mobility. Three authors (EMR, MP, CGMM) developed the structured questionnaires in English. Face validity was subsequently tested among four other authors (SJGG, MvdS, BV, RCIW) during several discussion rounds until consensus was reached. Both questionnaires were sent to Dutch researchers and clinicians between May to August 2021 working in the field of ageing and human movement sciences research and advertised through newsletters, on social media and through personal networks. All researchers and clinicians were encouraged to complete the questionnaire if their research and/or clinical practice was related to mobility. The questionnaires were developed and managed using REDCap (Research Electronic Data Capture) electronic data capture tools [20, 21]. The literature review results and the expert questionnaires were used to discuss and draft a proposition on the definition of mobility, conceptual framework, and constructs. Table 1 provides a glossary of used terms and their definitions. A waiver for ethical approval was obtained from the medical ethics committee of Amsterdam UMC, Amsterdam, The Netherlands. Completion of the questionnaire was taken as written informed consent. All procedures were performed in accordance with local and international ethical guidelines.

**Table 1:** Glossary

Term	Definition
Activity(-ies)	Execution of a task or action by an individual [15].
Body functions	Physiological and psychological functions of body systems [15].
Body structures	Anatomical parts of the body such as organs, limbs and their sub-structures [15].
Components	Components of health, health domains and health-related domains [15].
Conceptual framework	Network of interlinked concepts that together provide a comprehensive understanding of a phenomenon or phenomena [39].
Constructs	Abstract idea, underlying theme, or subject matter that one wishes to measure [40].
Domains	Practical and meaningful set of related physiological functions, anatomical structures, actions, tasks, or areas of life [15].
Environmental factors	Physical, social and attitudinal environment in which people live [15].
Measures	Proxy determinants or outcomes for constructs [41].
Participation	Involvement in a life situation [15].
Personal factors	Background of an individual’s life and living comprising features of the individual that are not part of a health condition or health states [15].

**Table 2:** Definitions of mobility reported in the literature

Reference	Definition
Guralnik et al., 1993 [26]	Ability to walk some distance and climb stairs.
WHO ICF, 2001 [15]	Moving by changing body position or location or by transferring from one place to another, by carrying, moving or manipulating objects, by walking, running or climbing, and by using various forms of transportation.
Routhier et al., 2003 [27]	Any movements that lead to a change in position or location by one’s own means performed with or without technical assistance.
Webber et al., 2010 [11]	Ability to move oneself (e.g., by walking, by using assistive devices, or by using transportation) within community environments that expand from one’s home, to the neighbourhood, and to regions beyond.
Prohaska et al., 2011 [25]	Ability of individuals to meet the challenges of the environment given their capabilities associated with movement within and between environments.
Satariano et al., 2012 [1]	Movement in all of its forms, including basic ambulation, transferring from a bed to a chair, walking for leisure and the completion of daily tasks, engaging in activities associated with work and play, exercising, driving a car, and using various forms of public transport.
Rosso et al., 2013 [28]	Ability of an individual to move about the environment.
Umstattd Meyer et al., 2014 [10]	Personal mobility: ability to perform activities of daily living, as measured through functional assessments (e.g., walking, standing, sitting, reaching, stooping, and so on), within a generic life space. Community mobility: recent driving history, limitations, and access to a vehicle, within a generic life space.
Soubra et al., 2019 [14]	The person’s ability to change his position or location or move from one place to another by walking and basic ambulation.

WHO: World Health Organization. ICF: International Classification of Functioning, Disability and Health

### Literature review: Reviewing definitions and conceptual frameworks on mobility

We broadly reviewed mobility definitions and conceptual frameworks, not limited to the physical domain of mobility. The electronic database PubMed was searched to identify relevant papers using a combination of the terms ‘mobility’, ‘definition’ and ‘framework’. Additional relevant papers were identified from reference searching and from reference sections of included papers. The search strategy was drafted by three authors (EMR, MP, CGMM); author EMR searched, selected and reviewed relevant papers. Outcomes of the search were discussed among authors EMR, MP, CGMM. Reported definitions of mobility and conceptual frameworks, i.e. conceptualisation, description, components and the aim of components (e.g. determinants, outcomes), were summarized in tables. Results of the literature review informed the development of the questionnaires.

### Questionnaire 1: Exploring the agreement on the definition of mobility, conceptual framework, constructs and measures of mobility

This questionnaire was developed to explore (dis)agreement and performing a needs evaluation to assess the need for a standardized definition, framework and constructs. The questionnaire consisted of six sections: respondent details, needs evaluation for mobility tools for researchers and/or clinicians, the definition of mobility, statements related to the definition of mobility, conceptual framework, constructs and measures of mobility, and the current use of constructs and measures. Respondent details encompassed age, gender, main position (research and/or clinical practice), years of research experience and the highest completed degree. The needs evaluation included questions on how mobility is used in the respondents’ research, i.e. participant characterization, determinant, primary outcome, secondary outcome, not measuring mobility (multiple choice), if a core-set of mobility measures should be used for mobility both as a determinant or outcome (five-point Likert scale), the use of a conceptual framework to assess mobility (yes/no) and if so which framework and if it is used in research and/or clinical practice (multiple choice). Furthermore, the needs evaluation included statements to assess the needs for mobility tools: a clear and standardized definition of mobility, a conceptual framework, an overview of constructs of mobility, a core-set of mobility measures, potential data sharing or use of other one’s data for mobility-related research. The definition of mobility was assessed as an open-ended question (‘how do you define mobility’). Statements were assessed using the five-point Likert scale from ‘strongly agree’ to ‘strongly disagree’ [22]. Open-ended questions were used to assess which constructs respondents were thinking of in the context of mobility and which measures they currently used in their research/clinical practice.

### Questionnaire 2: defining, classifying and identifying constructs

The second questionnaire was developed to allow for further clarification on constructs of mobility as informed by the results of questionnaire 1. The questionnaire consisted of four sections: respondent details, statements to assess the agreement on a conceptual framework and on defining constructs, the classification of constructs, and identification of constructs. Respondent details encompassed age, gender, main position (research and/or clinical practice), years of research experience and the highest completed degree. Statements (five-point Likert scale) were related to the use of the ICF framework and defining capacity, performance and function. The classification of constructs was assessed using multiple-choice questions using the ICF components [15] as answer options. The ICF components were used for the classification as the ICF was the most often used conceptual framework based on the results of questionnaire 1. The identification of 25 potential constructs was assessed on a scale of one to ten, with ten being the highest relevance.

### Data and statistical analysis

Descriptive statistics were used to present the results of quantitative questions. Categorical variables were presented as frequencies and percentages, normally distributed continuous variables as means and standard deviations (SD), and skewed continuous variables as medians and interquartile ranges (IQR). Agreement on statements (questionnaire 1 and 2) and the classification of constructs (questionnaire 2) was defined by combining the answer options ‘agree’ and ‘strongly agree’ and applying the following cut-offs expressed as the percentage of respondents: strong agreement ≥ 80%, moderate agreement ≥ 70% and < 80%, low agreement < 70% [23]. The open-ended question on the definition of mobility (questionnaire 1) was analysed by identifying themes using word repetitions (author EMR). The open-ended question on the constructs and measures of mobility were analysed by creating lists of all reported constructs and measures. Mobility measures were combined if they had the same overall categorization (e.g. Katz ADL). Results of open-ended questions were presented quantitatively by using frequencies and percentages. The identification of constructs (questionnaire 2) was analysed by the median scores with IQRs and by the percentage of respondents scoring eight, nine or ten and applying the cut-offs: very relevant ≥ 80%, moderately relevant ≥ 70% and < 80%, least relevant < 70% [23, 24]. Quantitative data were analysed using the Statistical Package for the Social Sciences (IBM SPSS Statistics for Windows, Version 27. Armonk, NY, IBM Corp). Qualitative data were analysed using Microsoft Excel (version 1808) (identification of themes and counts of reported constructs and measures).

## Results

### Literature review

#### Definitions of mobility

Nine relevant papers were identified, reporting various definitions of mobility (Table 2). Definitions overlapped regarding the terms movement and the ability to move and varied on conditions such as the forms of movement included, environment and the distinction of personal and community mobility. Definitions also varied regarding the inclusion [1, 11, 15, 25] of travel/commuting, not explicitly stating if travel/commuting was included [14, 26–28] or distinguished between mobility as the ability to move and mobility in terms of travel/commuting. Two definitions [14, 27] defined mobility similar to the ICF [15]. Another definition defined mobility as movement in all of its forms [1]. Webber et al. [11] defined mobility as the ability to move and includes different levels of life-spaces, from home to the neighbourhood and regions beyond, including travel/commuting. One definition distinguished between personal mobility, i.e. the ability to perform daily activities, and community mobility, i.e. travel/commuting [10]. Two definitions focused on the environment regarding the capacity or ability to move within and between environments [25, 28].

#### Conceptual frameworks

Seven conceptual frameworks of mobility were identified [9, 11, 15, 27, 29–31] of which three conceptualised mobility within broader frameworks and in four mobility was the primary concept [9, 11, 27, 31] (Table 3). Conceptual frameworks consisted of various components with different aims, i.e. a pathway [29], determinants and outcomes [15], assessment [30], influencing factors [27], risk factors [9] and determinants [11, 31].

**Table 3:** Conceptual frameworks of mobility reported in the literature

Reference	Conceptualisation	Description	Components	Aim components
*Conceptual frameworks in which mobility can be assessed within a broader framework*				
Verbrugge & Jette 1994 [29]	Disability: difficulty doing activities in any domain of life due to a health or physical problem.	The Disablement Process, a socio-medical model describes how chronic and acute conditions affect functioning in specific body systems, generic physical and mental actions, and activities of daily life, and describes the personal and environmental factors that speed or slow disablement, namely, risk factors, interventions, and exacerbators.	Concepts: ● Pathology ● Impairments (risk factors) ● Functional limitations: intra-individual factors (lifestyle & behaviour, psychosocial attributes & coping, activity accommodations) extra-individual factors (medical care & rehabilitation, medications & other therapeutic regimens, external supports, built, physical & social environment) ● Disability	Pathway from pathology to various kinds of functional outcomes
WHO ICF, 2001 [15]	Functioning: all body functions, activities and participation. Disability: impairments, activity limitations or participation restrictions. Health: a state of physical, mental and social well-being in which disease and infirmity are absent.	A classification of health and health-related domains, domains to describe changes in body function and structure, what a person with a health condition can do in a standard environment (their level of capacity), and what they actually do in their usual environment (their level of performance).	Components: ● Body functions and structures ● Activities and participation: capacity, performance ● Environmental factors ● Personal factors	Determinants and outcomes of health and health-related states
Tomey & Sowers, 2009 [30]	Physical functioning: being supported by physical abilities, as well as by those in the cognitive domain.	The PF-E conceptual model addresses the need for physical functioning assessments that reflect performance capacity, environmental factors, and coping and compensation strategies.	[not reported] ● Performance capacity ● Environmental factors (indoor and outdoor) ● Compensation/coping strategies	Assessment of physical functioning
***Conceptual frameworks primarily conceptualising mobility***				
Routhier et al. 2003 [27]	Mobility: any movements that lead to a change in position or location by one’s own means performed with or without technical assistance. Wheelchair mobility: being able to move the chair and use its accessories, such as the brakes or control interface.	Relational model of wheelchair mobility, a performance assessment framework, encompassing theoretical and conceptual views of wheelchair use found in various fields, particularly rehabilitation, social integration, occupational therapy, engineering, wheelchair design and sociology.	Categories of factors: ● Occupation and social participation ● Wheelchair (mechanical, electronic and ergonomic aspects) ● Assessment and training ● Daily activities and social roles ● Environment (physical dimension, socio-cultural dimension) ● User’s profile (medical & physical profiles, personality, attitude & temperament, socio-cultural relations & spirituality)	Influencing factors of mobility
Yeom et al. 2008 [9]	Mobility limitations: a state of impaired mobility, a condition in which an individual experience a limitation in independent physical movement, or is at risk for experiencing limitations.	An assessment guideline and intervention strategies for mobility limitations in older adults based on a social-ecological framework.	Factors: ● Intrapersonal factors: demographics, comorbid conditions, motivational, lifestyle and physiological factors ● Interpersonal factors: high interpersonal dependency, lack of social relations & participation ● Environmental factors: inconvenient indoor environment, lack of availability of services in local area, feeling of insecurity	Risk factors of mobility
Webber et al. 2010 [11]	Mobility: ability to move oneself (e.g., by walking, by using assistive devices, or by using transportation) within community environments that expand from one’s home, to the neighbourhood, and to regions beyond	Conical model illustrating seven life-space locations (world, surrounding area, service community, neighbourhood, outdoors, home, rooms), composing mobility determinants related to cognitive, psychosocial, physical, environmental, and financial factors. Gender, culture, and biographical influences exerting influence on all of the mobility determinants.	Categories of determinants: ● Financial ● Psychosocial ● Environmental ● Physical ● Cognitive ● Gender, Cultural and Biographical Influences	Determinants of mobility
Franke et al. 2020 [31]	Mobility: ability to move oneself (e.g., by walking, by using assistive devices, or by using transportation) within community environments that expand from one’s home, to the neighbourhood, and to regions beyond	An adapted mobility framework (from Webber et al. 2010) using a physiological, subjective, contextual and temporal approach, that provides a more comprehensive conceptualisation of the nature and processes of older adults’ mobility. The temporal approach reveals the dynamic, fluid and experiential nature of mobility by analysing factors within and between people and their environments, over time.	Concepts: ● Physiological: physical, chronic conditions, cognition ● Subjective: psychological, attitudes, perceptions ● Context: financial, built, social, natural environment, culture ● Temporal: factors within and between people and their environments, over time	Determinants of mobility

WHO: World Health Organization. ICF: International Classification of Functioning, Disability and Health. PF-E: Physical Functioning Assessment in Your Environment

Mobility was conceptualised within broader frameworks of disability [29], functioning, disability and health [15], and physical functioning [30]. One of the first frameworks is the Disablement Process framework, developed by Nagi in 1976 [32] and adapted by Verbrugge and Jette (1994) [29]. This framework is a socio-medical model and describes how chronic and acute conditions can affect functional outcomes [29]. It includes a pathway from pathology (diagnoses of disease, injury, congenital/developmental condition) to impairments (dysfunctions and structural abnormalities in specific body systems) to functional limitations (restrictions in basic physical and mental action) to disability (difficulty doing activities in daily life) and it distinguishes between intrinsic disability (without personal or equipment assistance) and actual disability (with such assistance) [29]. Physical actions in this framework include mobility and therefore, this framework is also used to assess mobility [29]. Another framework is the ICF encompassing components of health and health-related components of well-being to assess determinants and outcomes of health and health-related states by body functions and structures, activities and participation, environmental factors and personal factors [15]. The Physical Functioning Assessment in Your Environment (PF-E) framework was developed based on the ICF to assess physical functioning, reflecting performance capacity, environmental factors, and coping and compensation strategies [30]. Performance capacity is defined in this framework as diminished physical or cognitive ability, which includes mobility in a broad sense [30].

Frameworks primarily conceptualising mobility included a framework on wheelchair mobility [27], mobility limitations [9], different life-space locations and the complexity of determinants influencing mobility [11], and subjective and temporal elements of movement [31]. The wheelchair mobility framework is a relational model of factors influencing wheelchair mobility. It includes the components occupation and social participation, wheelchair mechanical, electronic and ergonomic aspects, assessment and training, daily activities and social roles, environment and the user’s medical and physical profile [27]. The social-ecological framework aims to understand mobility limitations on multiple levels encompassing risk factors of mobility, i.e. intrapersonal, interpersonal and environmental factors [9]. Webber et al.‘s framework includes multiple forms of movement and determinants influencing mobility [11]. It includes seven life-space locations (i.e. world, surrounding area, service community, neighbourhood, outdoors, home, rooms) and key determinants of mobility categorized under the domains financial, psychosocial, environmental, physical, cognitive, gender, cultural and biographical influences [11]. This framework was adapted to more comprehensively conceptualise the nature and processes of mobility using a temporal approach of factors within and between people and their environments over time, and by including subjective elements (i.e. psychological, attitudes, perceptions) next to the physiological and contextual components [31].

### Questionnaire 1

#### Respondent characteristics

A total of 66 respondents completed the questionnaire (**Additional** Table 3). The median age was 40 years (IQR 30–53) and 35 were females (53.0%). Forty-two respondents (63.6%) had worked mainly in research, 21 (31.8%) in research and clinical practice and three (4.5%) in clinical practice. The median of research experience was 11 years (IQR 4–23) and more than half of respondents (n = 36, 54.5%) had a doctorate degree as the highest completed degree. Respondents covered a range of research field/interest, including human movement (biomechanics, neuromechanics, gait), rehabilitation, surgery, lifestyle (exercise, nutrition), orthopaedics, physical activity, falls, bone and muscle.

#### Needs evaluation for mobility tools for researchers and/or clinicians

**Fig. 1:**
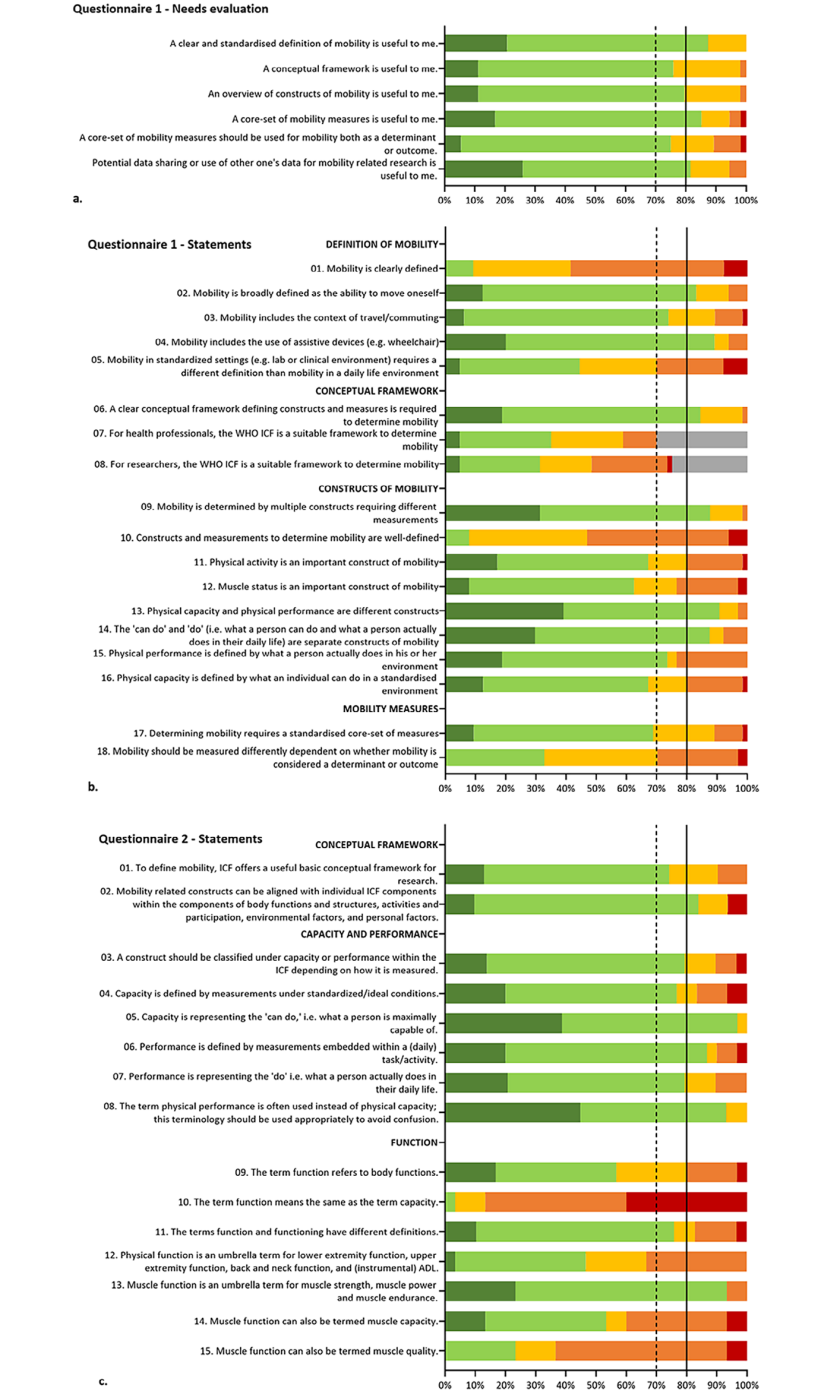
Visualization of the agreement on statements related to **(a)** needs evaluation for mobility tools for researchers and/or clinicians (questionnaire 1), **(b)** the definition of mobility, conceptual framework, constructs and measures of mobility (questionnaire 1) and **(c)** defining constructs (questionnaire 2) Colors indicate the answers on the five-point Likert scale: strongly agree (dark green), agree (light green), neither agree nor disagree (light orange), disagree (dark orange), strongly disagree (red), I do not have an opinion on this statement (grey). Agreement (strongly agree and agree) and disagreement (disagree and strongly disagree) cut-off values: strong: ≥80% (if the green bars pass the straight line), moderate ≥ 70% and < 80% (if the green bars pass the dotted line but not the straight line), low < 70% (if the green bars do not pass the dotted line)

Mobility was used as participant characterization (n = 28, 49.1%), determinant (n = 24, 42.1%), primary outcome (n = 31, 54.4%) and as a secondary outcome (n = 33, 57.9%). A third of respondents (n = 19, 33.9%) used a conceptual framework to assess mobility, with the ICF as the most common framework (n = 15). Frameworks were used with the purpose for combined research and clinical practice (n = 13), research (n = 5) and clinical practice (n = 1). Strong agreement was reached on the need for: a clear and standardized definition of mobility, a core-set of mobility measures, and potential data sharing or use of other one’s data for mobility-related research (Fig. 1a). Moderate agreement was reached on the usefulness of a conceptual framework, an overview of constructs of mobility, and that a core-set of mobility measures should be used for mobility both as a determinant or outcome (Fig. 1a).

#### Definition of mobility

Twenty-two themes were identified (**Additional** Table 4), with the most frequent themes being move/movement (n = 50, 75.8%) and the ability to move (n = 34, 51.5%). Other identified themes had lower frequencies and indicated the variety in reported definitions of mobility.

**Table 4:** Summary of the results and conclusions, and suggestions for (clinical) research

Summary results	Conclusions	Suggestions
Definition of mobility		
● Generally includes the terms movement and ability to move but varies on conditions. Strong agreement: ● A clear and standardised definition of mobility is useful to me. ● Mobility is broadly defined as the ability to move oneself. ● Mobility includes the use of assistive devices (e.g. wheelchair). Moderate agreement: ● Mobility includes the context of travel/commuting. Low agreement: ● Mobility is clearly defined. ● Mobility in standardized settings (e.g. lab or clinical environment) requires a different definition than mobility in a daily life environment.	● No uniform definition. ● Generally includes the terms movement and ability to move. ● Variability between definitions regarding the conditions.	● Define mobility as the ability to move, with or without the use of assistive devices. ● Specify conditions if any applied, i.e. the form of movement (e.g. walking, exercising, travel/ commuting), the role of the environment (e.g. ideal conditions, daily life).
**Conceptual framework**		
● Conceptual frameworks differ in how they conceptualise mobility and the included components. ● A third of respondents used a conceptual framework to assess mobility; the ICF was most often used. Strong agreement: ● A clear conceptual framework defining constructs and measures is required to determine mobility. ● Mobility related constructs can be aligned with individual ICF components within the components of body functions and structures, activities and participation, environmental factors, and personal factors. Moderate agreement: ● A conceptual framework is useful to me. ● To define mobility, ICF offers a useful basic conceptual framework for research. Low agreement: ● For health professionals, the WHO ICF is a suitable framework to determine mobility. ● For researchers, the WHO ICF is a suitable framework to determine mobility.	● Multiple conceptual frameworks are available in the literature with variability in how mobility is conceptualised. ● There is a need for a clear conceptual framework for mobility. ● There were conflicting opinions between both questionnaires on the suitability of the ICF as a framework to determine mobility.	● No recommendations can be made on a conceptual framework to determine the physical domain of mobility ● Use of the ICF framework to classify constructs in the components ‘body functions and structures’ and ‘activity and participation’, reflecting capacity and performance.
**Constructs**		
● Ninety-two unique constructs of mobility were reported. ● Median scores of the relevance of multiple constructs were similar (varied between 6 and 9]. ● Only ambulation, gait function, (instrumental) activities of daily living and physical activity were identified as moderately relevant ● None of the other constructs was identified as very or moderately relevant.	● Mobility encompasses multiple constructs. ● Variability in the classification and identification of constructs.	● Define if physical capacity and/or physical performance was assessed and how it was measured.
Strong agreement: ● Mobility is determined by multiple constructs requiring different measurements. ● Classification of muscle function under body functions and structures.	● Physical capacity and physical performance are different constructs with different definitions. ● Classification under capacity or performance depends on how it is measured.	● Physical capacity is defined by measurements under standardized/ideal conditions and represents the ‘can do,‘ i.e. what a person is maximally capable of.
● Physical capacity and physical performance are different constructs. ● The ‘can do’ and ‘do’ (i.e. what a person can do and what a person actually does in their daily life) are separate constructs of mobility. ● Capacity is representing the ‘can do,‘ i.e. what a person is maximally capable of. ● Performance is defined by measurements embedded within a (daily) task/activity. ● The term physical performance is often used instead of physical capacity; this terminology should be used appropriately to avoid confusion. ● The term function means the same as the term capacity. ● Muscle function is an umbrella term for muscle strength, muscle power and muscle endurance. Moderate agreement: ● An overview of constructs of mobility is useful to me. ● Classification of muscle quality under body functions and structures. ● Physical performance is defined by what a person actually does in his or her environment. ● A construct should be classified under capacity or performance within the ICF depending on how it is measured. ● Capacity is defined by measurements under standardized/ideal conditions. ● Performance is representing the ‘do’ i.e. what a person actually does in their daily life. ● The terms function and functioning have different definitions. Low agreement: ● Constructs and measurements to determine mobility are well-defined. ● Physical activity is an important construct of mobility. ● Muscle status is an important construct of mobility.	● Terminology of capacity and performance is used interchangeably. ● The term function is not defined and depends on what type of function is referred to (e.g. physical function, muscle function) ● The terms function and functioning differ in their definitions.	● Physical performance is defined by measurements embedded within a (daily) task/activity and represents the ‘do’ i.e. what a person actually does in their daily life. ● Define the terms function and functioning in terms of body functions, capacity or performance in relation to the type and environment of function(ing) assessed.
● Classification of all other constructs. ● Physical capacity is defined by what an individual can do in a standardised environment. ● The term function refers to body functions. ● Physical function is an umbrella term for lower extremity function, upper extremity function, back and neck function, and (instrumental) activities of daily living. ● Muscle function can also be termed muscle capacity. ● Muscle function can also be termed muscle quality.		● Constructs within the physical domain of mobility need to be further identified, defined and classified.
**Measures**		
● Eighty-nine unique measures of mobility were reported. Strong agreement: ● A core-set of mobility measures is useful to me. ● Potential data sharing or use of other one’s data for mobility related research is useful to me. Moderate agreement: ● A core-set of mobility measures should be used for mobility both as a determinant or outcome. Low agreement: ● Determining mobility requires a standardised core-set of measures. ● Mobility should be measured differently dependent on whether mobility is considered a determinant or outcome.	● Mobility is measured using multiple measures. ● A core-set of mobility is useful for researchers and/or clinicians, also in order to potentially share data in a standardised way.	● A core-set of measures should include the measure and the format of assessment to subsequently link them to the appropriate constructs.

WHO: World Health Organization. ICF: International Classification of Functioning, Disability and Health

#### Agreement on the definition of mobility, conceptual framework, constructs and measures of mobility within the physical domain of mobility

Strong agreement (Fig. 1b, **Additional Table 5**) was reached on six statements: two on the definition of mobility, one on conceptual frameworks and three on mobility constructs. Moderate agreement was reached on two statements: one on the definition of mobility and one on mobility constructs. None of the statements on measures of mobility reached strong or moderate (dis)agreement. Two statements on mobility measures were not depicted in Fig. 1. Gait speed measured over a four-meter course was reported as a physical capacity measure (n = 28, 44.4%), a physical performance measure (n = 24, 38.1%) and both a physical capacity and performance measure (n = 11, 17.5%). ADL was reported as a physical capacity measure (n = 8, 12.7%), a physical performance measure (n = 35, 55.6%), and both a physical capacity and performance measure (n = 20, 31.7%).

#### Constructs and measures of mobility within the physical domain of mobility

Ninety-two unique mobility constructs were reported out of 157 reported constructs with a median frequency of 1 [IQR 1–2] (**Additional Table 6**). Constructs reported above the median encompassed: physical activity (n = 10), assistive devices (n = 8), ADL (n = 6), ability to move (n = 5), balance (n = 5), walking/ability to walk (n = 5), capacity (n = 4), joint (function, mobility, range of motion) (n = 4), movement (n = 4), performance (n = 4), physical function (n = 4), health (status) (n = 3), muscle strength (n = 3), physical capacity (n = 3), climbing stairs (n = 3), cognition (n = 2), environment (n = 2), exercise capacity (n = 2), mental health (n = 2), motivation (n = 2), muscle function (n = 2), participation (n = 2), quality of movement (n = 2), range of motion (n = 2) and transportation (n = 2).

Eighty-nine unique mobility measures were reported out of 172 reported measures with a median frequency of 1 [IQR 1–1] (**Additional Table 7**). Measures reported above the median encompassed: physical activity (n = 19), walking test (n = 18), muscle strength (n = 9), Short Physical Performance Battery (SPPB) (n = 8), ADL (n = 7), balance (n = 6), gait quality (n = 4), aids (n = 3), Activity Measure for Post-Acute Care (AM-PAC) (n = 3), chair stand (n = 3), de Morton Mobility Index (DEMMI) (n = 3), range of motion (n = 3), step test (n = 3), adaptability of gait (n = 2), capacity (n = 2), muscle function (n = 2), performance (n = 2), physical functioning (n = 2), physical performance (n = 2), and Timed Up and Go (TUG) (n = 2).

### Questionnaire 2

#### Respondent characteristics

A total of 31 respondents completed the questionnaire with similar characteristics compared to respondents of questionnaire 1 (**Additional** Table 3). The median age was 39 years (IQR 30–52) and 16 were females (51.6%). Twenty-five respondents (80.6%) had worked mainly in research, 4 (12.9%) in research and clinical practice and two (6.5%) in clinical practice. The median research experience was 15 years (IQR 4–25) and 20 respondents (64.5%) had a doctorate degree as the highest completed degree.

#### Agreement on defining constructs

Strong agreement was reached on six statements (Fig. 1c and **Additional Table 5**): one on conceptual frameworks, three on capacity and performance and two on function. Moderate agreement was reached on five statements: one on conceptual frameworks, three on capacity and performance and one on function.

#### Classifying constructs using the ICF components

A strong agreement was found for classifying muscle function under body functions and structures; a moderate agreement was found for classifying muscle quality under body functions and structures (**Additional Table 8**). Low agreement was found for all other classifications of constructs.

#### Identifying constructs of mobility

**Fig. 2:**
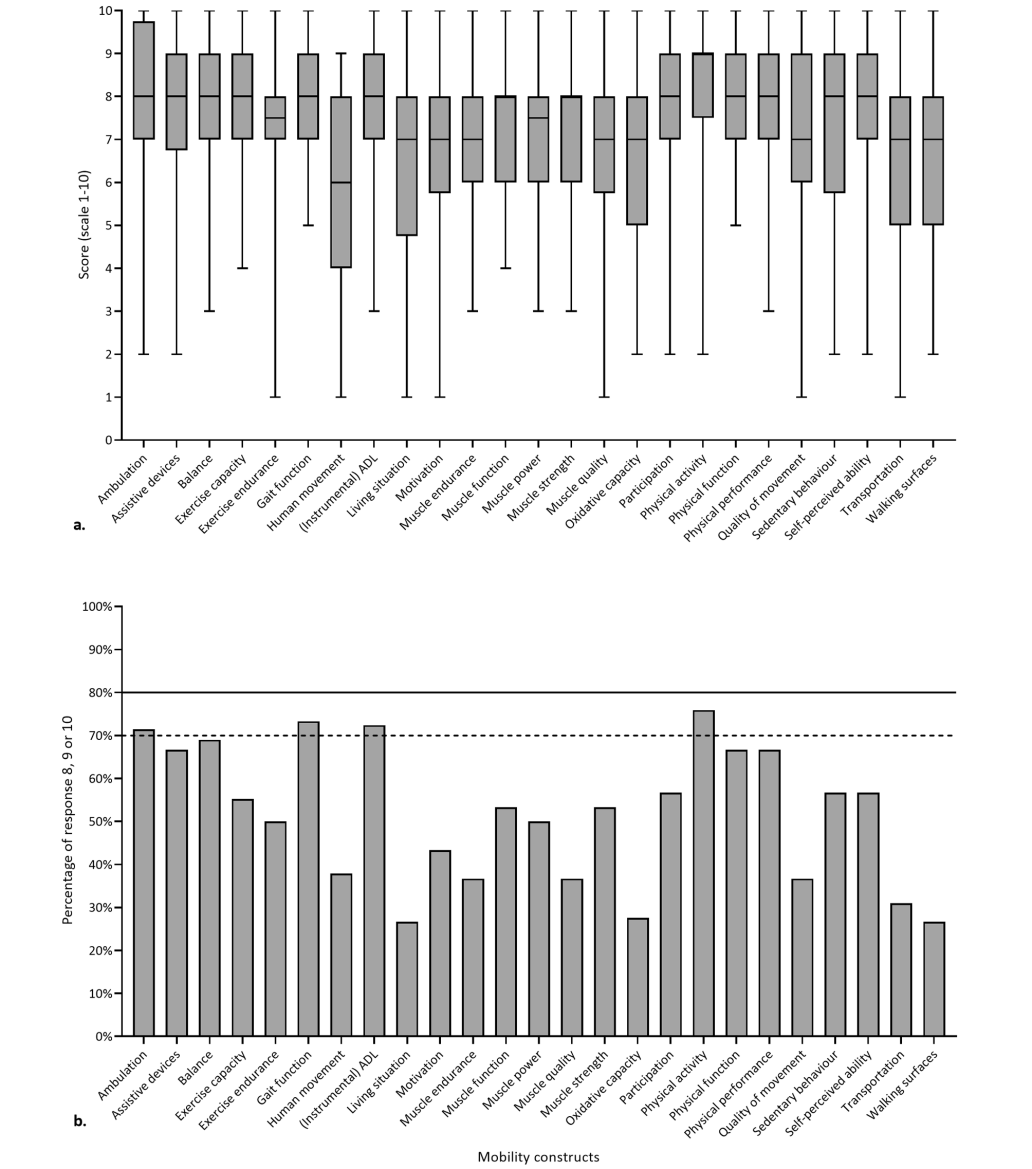
Identification of constructs of mobility, assessed in questionnaire 2 (defining, classifying and identifying constructs) Data is presented as **(a)** the scoring on the scale of 1–10 with 10 being the highest relevance using box and whisker plots and as **(b)** the percentage of respondents scoring 8, 9 or 10 ADL: activities of daily living. Relevance cut-off values: very relevant ≥ 80% (if the bar pass the straight line), moderately relevant ≥ 70% and < 80% (if the bar pass the dotted line but not the straight line), least relevant < 70% (if the bar does not pass the dotted line)

Median scores ranged from 6 to 9 points (Fig. 2a). Ambulation, gait function, (instrumental) ADL and physical activity were identified as moderately relevant (Fig. 2b). None of the other constructs was identified as very or moderately relevant.

## Discussion

Standardization, data comparison and data sharing require uniform core-sets of measurements addressing distinct constructs of mobility aligned with conceptual frameworks, considering the multifactorial nature of mobility. In this paper, we aimed to review and assess the agreement on definitions, conceptual frameworks and constructs of mobility in ageing research. We found strong expert agreement on the usefulness of a core-set of mobility measures and potential data sharing or use of other one’s data for mobility-related research. While a variety of definitions and conceptual frameworks of mobility were identified in the literature, we found strong agreement amongst researchers and clinicians on defining mobility as the ability to move, including the use of assistive devices. We also found variability in the literature on how mobility was conceptualised within frameworks and a strong expert agreement that a clear conceptual framework defining constructs and measures is required to determine mobility. However, we found conflicting opinions between both questionnaires on the suitability of the ICF as a framework to determine mobility. Within the physical domain, we found strong agreement that mobility is determined by multiple constructs requiring different measurements and to distinguish the ‘can do’ and ‘do’ as separate constructs of mobility, with strong agreement that these constructs are defined by physical capacity and physical performance, respectively. Function and functioning are not well-defined and clearly discriminated, despite the strong agreement that these terms have different definitions. Lastly, we found low agreement on the classification of constructs and variability in identifying relevant constructs. Table 4 summarizes our results, conclusions and provides suggestions for (clinical) research.

### Definition of mobility

Mobility was generally defined in the literature as the ability to move with or without assistive devices. This was also reflected by a strong agreement among respondents. Variability in definitions was mainly related to the conditions such as the form of movement, e.g. walking, exercising, travel/commuting, and the role of the environment, e.g. ideal conditions, daily life. This underpins its multifactorial nature and the need to define conceptual frameworks. Definitions from the literature also differed regarding the inclusion or exclusion of travel/commuting, which was reflected by a moderate agreement to include the context of travel/commuting within the definition. Differences between definitions makes it difficult to compare mobility between studies and disciplines within the physical domain. We propose to define mobility as the ability to move, with or without assistive devices. Researchers and clinicians should align and/or clearly specify their definition as well as any conditions applied.

### Conceptual framework

None of the multiple conceptual frameworks for mobility described in the literature focus primarily on the physical domain of mobility. Most frameworks encompass multiple components/domains to highlight interactions between these different components/domains [9, 11, 27, 31]. Furthermore, these components/domains aim to assess determinants, risk factors or influencing factors of mobility and do not consider mobility as an outcome. We found low agreement to measure mobility differently depending on whether mobility is considered a determinant or outcome and moderate agreement for a core-set of measures for mobility both as a determinant or outcome. These results indicate that mobility is a concept that can be used both as a determinant (e.g. of disability) and outcome.

A clear conceptual framework defining constructs and measures focussed on the physical domain of mobility is highly relevant in ageing research as was confirmed with strong agreement by the respondents. Although there was no agreement on the usability of the ICF in clinical and fundamental research, the ICF framework offers the possibility to focus on the physical domain of mobility and to classify constructs in the components ‘body functions and structures’, ‘activity’ and ‘participation’, reflecting capacity (i.e. executing tasks in a standard environment) and performance (i.e. executing tasks in the current environment), which was also recognized with strong expert agreement. The ICF framework is already often used clinically, especially in rehabilitation [33], and was also the most often used framework for mobility among respondents. This makes it easier for communication and implementation, albeit its challenges, particularly the non-standard use of capacity and performance with varying interpretations of definitions and the role of the environment (i.e. standardised and current environment [34].

### Constructs of mobility

Two important and widely used constructs for mobility are capacity and performance. The ICF differentiates both constructs by the environment of assessment and defines capacity as the ability to execute a task or an action in a standard environment and performance what an individual does in the current environment [15]. It is important to note that the terms capacity and performance within the ICF are broad terms and can be used in relation to multiple domains. Within the physical domain, respondents strongly agreed that physical capacity and physical performance are different constructs, although the terms are often used inappropriately and interchangeably. For example, mobility assessments in a standardised environment, such as gait speed on the four-meter walk test [35], is often coined physical performance, while it rather is a capacity measure according to the ICF definitions. Gait speed can also be measured in daily life using accelerometers [36], however, these two different ways of measuring gait speed are weakly correlated [36] as they measure different constructs. Gait speed by the four-meter walk test represents the ‘can do’ and therefore the physical capacity construct, while gait speed by accelerometers represents the ‘do’ and therefore the physical performance construct. Another example is the assessment of ADL, which is also dependent on how it is assessed leading to discrepancies between the ‘can do’ and ‘do’ ways of measuring [37]. Our results also reflected this difference as respondents differed in their opinion regarding gait speed and ADL as physical capacity and/or physical performance measures. In line with the difference in the format of assessment, a strong agreement was found regarding the distinction between the ‘can do’ and ‘do’ (i.e. what a person can do and what a person actually does in their daily life, respectively) when determining mobility. We therefore propose to define physical capacity by measurements under standardized/ideal conditions representing the ‘can do’ (i.e. what a person is maximally capable of) and physical performance by measurements embedded within a (daily) task/activity representing the ‘do’ (i.e. what a person actually does in their daily life).

Other often used construct terms are ‘function’ and ‘functioning’. The term functioning is defined within the ICF as an umbrella term for all body functions, activities and participation, and therefore including capacity and performance [15]. The term function itself is not defined and seems to depend on what type of function is referred to, such as physical function and muscle function. This also resulted in low agreement among respondents on definitions of physical function and muscle function. We found low agreement on function referring to body functions as defined by the ICF [15], but strong agreement on function having a similar meaning as capacity. Moreover, a moderate agreement was found on the terms function and functioning reflecting different definitions. We propose that in ageing research, the terms function and functioning are further clarified, e.g. whether body functions, capacity or performance and the type and environment of function(ing) are assessed.

Respondents further identified multiple mobility constructs within the physical domain of mobility, with comparable scores on the relevance of each of these constructs. Nonetheless, none of the constructs was identified as very relevant and only a few as moderately relevant when applying cut-offs. Furthermore, there was also low agreement on the classification of constructs under the ICF components. Although respondents indicated that an overview of mobility of constructs within the physical domain would be useful in (clinical) research, wide variability in mobility constructs resulted from specific research questions or clinical problems addressed [18]. In order to define a core-set of measures, constructs within the physical domain of mobility need to be identified and defined to link mobility measures subsequently. Further steps are required to reach a consensus on mobility constructs, such as a Delphi process [38].

In our appeal for the standardisation of mobility measures to facilitate ageing research, we also found that respondents used multiple measures to assess mobility, which could also be related to the use of multiple constructs. There is also variability in the type and purpose of measures in the literature and the format of assessment [14]. The format of assessment, i.e. in which conditions something is measured, reflects what the measure is assessing, e.g. body functions or structures, capacity or performance. A core-set of measures should not only take the measure (e.g. gait speed) into account but more importantly, the format of assessment to subsequently link them to the appropriate constructs.

### Strengths and limitations

This study is the first to comprehensively review the conceptualisation of mobility combining findings from the literature and exploring agreement among researchers and clinicians next to a needs evaluation to identify if the end-users also acknowledge the gaps in the literature. A limitation is that this study did not aim to include a formal process to reach consensus but rather explored the current agreement and/or discordance among experts as a first step. Due to the nature of questionnaires, there could have been selection bias in who completed the questionnaire(s), e.g. Dutch experts only and interested experts. Furthermore, limited data was available on the background and expertise of respondents.

## Conclusion

With the understanding that standardised measures of mobility require clear definitions and uniformity of conceptual frameworks and constructs, we observed the use of multiple constructs and measures that vary their definitions, classifications, and relevance. Based on our findings, we propose defining mobility as the ability to move, with or without assistive devices. Mobility constructs and measures in the physical domain should be classified appropriately under the conditions assessed. This represents what a person is maximally capable of (‘can do’) as capacity or what a person actually does in their current environment (‘do’) as performance. A framework like the WHO ICF allows for (clinical) classification of mobility constructs within the components of body functions and structures (capacity) and activities (performance).However, at this stage it is too early to recommend the ICF as the framework of choice. As this study was of exploratory nature, further steps are required to reach consensus within a systematic approach such as a Delphi process on a conceptual framework to determine mobility and to identify, define and classify mobility constructs. This will allow for a core-set of measures aligned to appropriate constructs to facilitate communication, interpretation and standardisation, following the Findability, Accessibility, Interoperability, and Reuse (FAIR) data principles [19], in operationalising the physical domain of mobility. Our results can inform next steps to systematically approach the definition, frameworks and constructs of mobility and to conduct a formal consensus procedure.

## Electronic supplementary material

Below is the link to the electronic supplementary material.

**Additional Table 1**. Questionnaire 1: Exploring the agreement on the definition of mobility, conceptual framework, constructs and measures of mobility. **Additional Table 2**. Questionnaire 2: Defining, classifying and identifying constructs. **Additional Table 3**. Characteristics of respondents to questionnaire 1 (n=66) and questionnaire 2 (n=31). **Additional Table 4**. Themes identified in the definition of mobility responses (n=66), assessed in questionnaire 1. **Additional Table 5**. Agreement on statements related to the definition of mobility, conceptual framework, constructs and measures of mobility (questionnaire 1) and defining constructs (questionnaire 2). **Additional Table 6**. Reported constructs of mobility, assessed in questionnaire 1. **Additional Table 7**. Reported measures of mobility, assessed in questionnaire 1. **Additional Table 8**. Assessment of classification of constructs using the ICF components, assessed in questionnaire 2

## Data Availability

The datasets used and/or analysed during the current study are available from the corresponding author on reasonable request.

## List of Abbreviations

ADL: Activities of daily living
AM-PAC: Activity Measure for Post-Acute Care
DEMMI: de Morton Mobility Index.
FAIR: Findability, Accessibility, Interoperability, and Reuse.
ICF: International Classification of Functioning, Disability and Health.
IQR: Interquartile range.
PF-E: Physical Functioning Assessment in Your Environment.
REDCap: Research Electronic Data Capture.
SD: Standard deviation.
SPPB: Short Physical Performance Battery.
TUG: Timed Up and Go.
WHO: World Health Organization.

## References

[1] Satariano WA, Guralnik JM, Jackson RJ, Marottoli RA, Phelan EA, Prohaska TR (2012). Mobility and aging: new directions for public health action. Am J Public Health.

[2] Shumway-Cook A, Ciol MA, Yorkston KM, Hoffman JM, Chan L (2005). Mobility limitations in the Medicare population: prevalence and sociodemographic and clinical correlates. J Am Geriatr Soc.

[3] Hirvensalo M, Rantanen T, Heikkinen E (2000). Mobility difficulties and physical activity as predictors of mortality and loss of independence in the community-living older population. J Am Geriatr Soc.

[4] von Bonsdorff M, Rantanen T, Laukkanen P, Suutama T, Heikkinen E (2006). Mobility limitations and cognitive deficits as predictors of institutionalization among community-dwelling older people. Gerontology.

[5] Fagerstrom C, Borglin G (2010). Mobility, functional ability and health-related quality of life among people of 60 years or older. Aging Clin Exp Res.

[6] Olaya B, Moneta MV, Domenech-Abella J, Miret M, Bayes I, Ayuso-Mateos JL (2018). Mobility difficulties, physical activity, and all-cause mortality risk in a nationally representative sample of older adults. J Gerontol A Biol Sci Med Sci.

[7] Gordt K, Paraschiv-Ionescu A, Mikolaizak AS, Taraldsen K, Mellone S, Bergquist R (2020). The association of basic and challenging motor capacity with mobility performance and falls in young seniors. Arch Gerontol Geriatr.

[8] Montero-Odasso M, Almeida QJ, Bherer L, Burhan AM, Camicioli R, Doyon J (2019). Consensus on Shared Measures of mobility and cognition: from the Canadian Consortium on Neurodegeneration in Aging (CCNA). J Gerontol A Biol Sci Med Sci.

[9] Yeom HA, Fleury J, Keller C (2008). Risk factors for mobility limitation in community-dwelling older adults: a social ecological perspective. Geriatr Nurs.

[10] Umstattd Meyer MR, Janke MC, Beaujean AA (2014). Predictors of older adults’ personal and community mobility: using a comprehensive theoretical mobility framework. Gerontologist.

[11] Webber SC, Porter MM, Menec VH (2010). Mobility in older adults: a comprehensive framework. Gerontologist.

[12] Metz DH (2000). Mobility of older people and their quality of life. Transp Policy.

[13] Anderson LA, Slonim A, Yen IH, Jones DL, Allen P, Hunter RH (2014). Developing a framework and priorities to promote mobility among older adults. Health Educ Behav.

[14] Soubra R, Chkeir A, Novella JL (2019). A systematic review of thirty-one Assessment tests to evaluate mobility in older adults. Biomed Res Int.

[15] World Health Organization. International classification of functioning, disability and health: ICF. World Health Organization. ; 2001. Available from: https://apps.who.int/iris/handle/10665/42407.

[16] Lamb SE, Keene DJ (2017). Measuring physical capacity and performance in older people. Best Pract Res Clin Rheumatol.

[17] Rojer AGM, Coni A, Mellone S, Van Ancum JM, Vereijken B, Helbostad JL, et al. Robustness of In-Laboratory and daily-life gait speed measures over one year in high functioning 61- to 70-Year-old adults. Gerontology. 2021;1–10. 10.1159/000514150. 10.1159/000514150 33752214

[18] Bussmann JB, Stam HJ (1998). Techniques for measurement and assessment of mobility in rehabilitation: a theoretical approach. Clin Rehabil.

[19] Wilkinson MD, Dumontier M, Aalbersberg IJ, Appleton G, Axton M, Baak A (2016). The FAIR Guiding Principles for scientific data management and stewardship. Sci Data.

[20] Harris PA, Taylor R, Minor BL, Elliott V, Fernandez M, O’Neal L (2019). The REDCap consortium: building an international community of software platform partners. J Biomed Inform.

[21] Harris PA, Taylor R, Thielke R, Payne J, Gonzalez N, Conde JG (2009). Research electronic data capture (REDCap)- -a metadata-driven methodology and workflow process for providing translational research informatics support. J Biomed Inform.

[22] Preedy V, Watson R, Watson RR (2010). 5-point likert scale. Handbook of Disease Burdens and Quality of Life Measures.

[23] Hsu C-C, Sandford BA (2007). The Delphi technique: making sense of consensus. Practical Assess Res Evaluation.

[24] Rodriguez-Manas L, Feart C, Mann G, Vina J, Chatterji S, Chodzko-Zajko W et al. Searching for an operational definition of frailty: a Delphi method based consensus statement: the frailty operative definition-consensus conference project. J Gerontol A Biol Sci Med Sci. 2013;68(1):62 – 7. 10.1093/gerona/gls119. 10.1093/gerona/gls119 PMC3598366 22511289

[25] Prohaska TR, Anderson LA, Hooker SP, Hughes SL, Belza B (2011). Mobility and aging: transference to transportation. J Aging Res.

[26] Guralnik JM, LaCroix AZ, Abbott RD, Berkman LF, Satterfield S, Evans DA (1993). Maintaining mobility in late life. I. demographic characteristics and chronic conditions. Am J Epidemiol.

[27] Routhier F, Vincent C, Desrosiers J, Nadeau S (2003). Mobility of wheelchair users: a proposed performance assessment framework. Disabil Rehabil.

[28] Rosso AL, Studenski SA, Chen WG, Aizenstein HJ, Alexander NB, Bennett DA (2013). Aging, the central nervous system, and mobility. J Gerontol A Biol Sci Med Sci.

[29] Verbrugge LM, Jette AM (1994). The disablement process. Soc Sci Med.

[30] Tomey KM, Sowers MR (2009). Assessment of physical functioning: a conceptual model encompassing environmental factors and individual compensation strategies. Phys Ther.

[31] Franke T, Sims-Gould J, Chaudhury H, Winters M, McKay H (2020). Re-framing mobility in older adults: an adapted comprehensive conceptual framework. Qual Res Sport Exerc Health.

[32] Nagi SZ (1976). An epidemiology of disability among adults in the United States. Milbank Mem Fund Q Health Soc.

[33] Cerniauskaite M, Quintas R, Boldt C, Raggi A, Cieza A, Bickenbach JE (2011). Systematic literature review on ICF from 2001 to 2009: its use, implementation and operationalisation. Disabil Rehabil.

[34] Jelsma J (2009). Use of the International classification of Functioning, disability and health: a literature survey. J Rehabil Med.

[35] Guralnik JM, Simonsick EM, Ferrucci L, Glynn RJ, Berkman LF, Blazer DG (1994). A short physical performance battery assessing lower extremity function: association with self-reported disability and prediction of mortality and nursing home admission. J Gerontol.

[36] Van Ancum JM, van Schooten KS, Jonkman NH, Huijben B, van Lummel RC, Meskers CGM (2019). Gait speed assessed by a 4-m walk test is not representative of daily-life gait speed in community-dwelling adults. Maturitas.

[37] Bootsma-van der Wiel A, Gussekloo J, de Craen AJ, van Exel E, Knook DL, Lagaay AM (2001). Disability in the oldest old: “can do” or “do do”?. J Am Geriatr Soc.

[38] Humphrey-Murto S, Varpio L, Wood TJ, Gonsalves C, Ufholz LA, Mascioli K (2017). The Use of the Delphi and other Consensus Group Methods in Medical Education Research: a review. Acad Med.

[39] Jabareen Y (2009). Building a conceptual Framework: Philosophy, definitions, and Procedure. Int J Qual Methods.

[40] Lavrakas PJ (2008). Encyclopedia of Survey Research Methods. Sage Publications.

[41] Miller VA, Reynolds WW, Ittenbach RF, Luce MF, Beauchamp TL, Nelson RM (2009). Challenges in measuring a new construct: perception of voluntariness for research and treatment decision making. J Empir Res Hum Res Ethics.

