# Supplementary material for: Towards a core-set of mobility measures in ageing research: The need to define mobility and its constructs
Source: BMC Geriatr. 2023 Apr 6;23:220. doi: 10.1186/s12877-023-03859-5 (PMC10080758; doi:10.1186/s12877-023-03859-5)
Supplement: Supplementary file 1 — Additional Table 1. Questionnaire 1: Exploring the agreement on the definition of mobility, conceptual framework, constructs and measures of mobility. Additional Table 2. Questionnaire 2: Defining, classifying and identifying constructs. Additional Table 3. Characteristics of respondents to questionnaire 1 (n=66) and questionnaire 2 (n=31). Additional Table 4. Themes identified in the definition of mobility responses (n=66), assessed in questionnaire 1. Additional Table 5. Agreement on statements related to the definition of mobility, conceptual framework, constructs and measures of mobility (questionnaire 1) and defining constructs (questionnaire 2). Additional Table 6. Reported constructs of mobility, assessed in questionnaire 1. Additional Table 7. Reported measures of mobility, assessed in questionnaire 1. Additional Table 8. Assessment of classification of constructs using the ICF components, assessed in questionnaire 2 [file 12877_2023_3859_MOESM1_ESM.docx]

**Additional files**

**Additional Table 1.** Questionnaire 1: Exploring the agreement on the definition of mobility, conceptual framework, constructs and measures of mobility

**Additional Table 2.** Questionnaire 2: Defining, classifying and identifying constructs

**Additional Table 3.** Characteristics of respondents to questionnaire 1 (n=66) and questionnaire 2 (n=31)

**Additional Table 4.** Themes identified in the definition of mobility responses (n=66), assessed in questionnaire 1

**Additional Table 5.** Agreement on statements related to the definition of mobility, conceptual framework, constructs and measures of mobility (questionnaire 1) and defining constructs (questionnaire 2)

**Additional Table 6.** Reported constructs of mobility, assessed in questionnaire 1

**Additional Table 7.** Reported measures of mobility, assessed in questionnaire 1

**Additional Table 8.** Assessment of classification of constructs using the ICF components, assessed in questionnaire 2

**Additional Table 1.** Questionnaire 1: Exploring the agreement on the definition of mobility, conceptual framework, constructs and measures of mobility

| **Question** | **Answer option(s)** |
| --- | --- |
| **Respondent details** |  |
| Age (years) | Open-ended |
| Gender | Single answer:   - Male - Female - Other - Prefer not to say |
| What is your main position in? | Single answer:   - Research - Clinical practice - Research and clinical practice |
| If you are working in clinical practice, please specify your profession | Open-ended |
| How many years research experience do you have? | Open-ended |
| What is the highest degree you have completed? | Single answer:   - Associate degree - Bachelor’s degree - Master’s degree - Professional degree (e.g. MD) - Doctorate degree |
| **Needs evaluation for mobility tools** |  |
| What role does mobility play in your research? | Single answer:   - Participant characterization - Determinant - Primary outcome - Secondary outcome - I do not measure mobility in my research |
| Do you use a conceptual framework or model to assess mobility (e.g. ICF)? | Single answer:   - Yes - No |
| If yes, which conceptual framework or model do you use? | Open-ended |
| If yes, do you use this conceptual framework or model for research and/or clinical practice? | Single answer:   - Research - Clinical practice - Research and clinical practice |
|  |  |
| A clear and standardized definition of mobility is useful to me. | Single answer:   - Strongly disagree - Disagree - Neither agree nor disagree - Agree - Strongly agree |
| A conceptual framework is useful to me. |  |
| An overview of constructs of mobility is useful to me. |  |
| A core-set of mobility measures is useful to me. |  |
| A core-set of mobility measures should be used for mobility both as a determinant or outcome. |  |
| Potential data sharing or use of other one's data for mobility related research is useful to me. |  |
| If you think about mobility, you think of the following constructs: | Open-ended |
| What key measures of mobility do you use in your research? | Open-ended |
| **Definition of mobility** |  |
| How do you define mobility? | Open-ended |
| **Statements on the definition of mobility, conceptual framework, constructs and measures of mobility** |  |
| Mobility is clearly defined. | Single answer:   - Strongly disagree - Disagree - Neither agree nor disagree - Agree - Strongly agree |
| Mobility is broadly defined as the ability to move oneself. |  |
| Mobility includes the context of travel/commuting. |  |
| Mobility includes the use of assistive devices (e.g. wheelchair). |  |
| Mobility in standardized settings (e.g. lab or clinical environment) requires a different definition than mobility in a daily life environment. |  |
| A clear conceptual framework defining constructs and measures is required to determine mobility. |  |
| For health professionals, the WHO ICF is a suitable framework to determine mobility. |  |
| For researchers, the WHO ICF is a suitable framework to determine mobility. |  |
| Mobility is determined by multiple constructs requiring different measurements. |  |
| Constructs and measurements to determine mobility are well-defined. |  |
| Physical activity is an important construct of mobility. |  |
| Muscle status is an important construct of mobility. |  |
| Physical capacity and physical performance are different constructs. |  |
| The 'can do' and 'do' (i.e. what a person can do and what a person actually does in their daily life) are separate constructs of mobility. |  |
| Physical performance is defined by what a person actually does in his or her environment. |  |
| Physical capacity is defined by what an individual can do in a standardised environment. |  |
| Determining mobility requires a standardised core-set of measures. |  |
| Mobility should be measured differently dependent on whether mobility is considered a determinant or outcome. |  |
| Gait speed measured over a four-meter course is a measure of: | Single answer:   - Physical performance - Physical capacity - Physical performance and physical capacity |
| Activities of daily living (ADL) is a measure of: |  |

WHO: World Health Organization. ICF: International Classification of Functioning, Disability and Health.

**Additional Table 2.** Questionnaire 2: Defining, classifying and identifying constructs

| **Question** | **Answer option(s)** |
| --- | --- |
| **Respondent details** |  |
| Age (years) | Open-ended |
| Gender | Single answer:   - Male - Female - Other - Prefer not to say |
| What is your main position in? | Single answer:   - Research - Clinical practice - Research and clinical practice |
| If you are working in clinical practice, please specify your profession | Open-ended |
| How many years research experience do you have? | Open-ended |
| What is the highest degree you have completed? | Single answer:   - Associate degree - Bachelor’s degree - Master’s degree - Professional degree (e.g. MD) - Doctorate degree |
| **Statements to define constructs** |  |
| To define mobility, ICF offers a useful basic conceptual framework for research. | Single answer:   - Strongly disagree - Disagree - Neither agree nor disagree - Agree - Strongly agree |
| Mobility related constructs can be aligned with individual ICF components within the components of: body functions and structures, activities and participation, environmental factors, and personal factors. |  |
| A construct should be classified under Capacity or Performance within the ICF depending on how it is measured. |  |
| Capacity is defined by measurements under standardized/ideal conditions. |  |
| Capacity is representing the 'can do,' i.e. what a person is maximally capable of. |  |
| Performance is defined by measurements embedded within a (daily) task/activity. |  |
| Performance is representing the 'do' i.e. what a person actually does in their daily life. |  |
| The term physical performance is often used instead of physical capacity; this terminology should be used appropriately to avoid confusion. | Single answer:   - Strongly disagree - Disagree - Neither agree nor disagree - Agree - Strongly agree |
| The term function refers to body functions. |  |
| The term function means the same as the term capacity. |  |
| The terms function and functioning have different definitions. |  |
| Physical function is an umbrella term for lower extremity function, upper extremity function, back and neck function, and (instrumental) activities of daily living. |  |
| Muscle function is an umbrella term for muscle strength, muscle power and muscle endurance. | Single answer:   - Strongly disagree - Disagree - Neither agree nor disagree - Agree - Strongly agree |
| Muscle function can also be termed muscle capacity. |  |
| Muscle function can also be termed muscle quality. |  |
| **Classifying constructs** |  |
| Functions should be classified under: | Single answer:   - Body functions and structures - Activities and participation – Capacity - Activities and participation – Performance - All of the above, depending on what and how it is measured - Unsure |
| Functioning should be classified under: |  |
| Physical function is: | Single answer:   - Another word for mobility with the same definition - A construct within: body functions and structures - A construct within: activities and participation - capacity - A construct within: activities and participation - performance - A separate construct within activities and participation, next to capacity and performance - Unsure |
| Human movement is: |  |
| Ambulation is: |  |
| Muscle function should be classified under: | Single answer:   - Body functions and structures - Activities and participation – Capacity - Activities and participation – Performance - Activities and participation - Capacity & Performance - Unsure |
| Muscle quality should be classified under: |  |
| Quality of movement should be classified under: |  |
| Gait function should be classified under: |  |
| Balance should be classified under: |  |
| (Instrumental) activities of daily living should be classified under: |  |
| Exercise capacity should be classified under: |  |
| Oxidative capacity should be classified under: |  |
| Exercise endurance should be classified under: |  |
| Muscle endurance should be classified under: | Single answer:   - Exercise endurance - Muscle capacity - Separate construct - Unsure |
| **Identifying constructs** |  |
| Ambulation, Assistive devices, Balance, Exercise capacity, Exercise endurance, Gait function, Human movement, (Instrumental) activities of daily living, Living situation, Motivation, Muscle endurance, Muscle function, Muscle power, Muscle strength, Muscle quality, Oxidative capacity, Participation, Physical activity, Physical function, Physical performance, Quality of movement, Sedentary behaviour, Self-perceived ability, Transportation, Walking surfaces | Single answer:   - Scale 1-10 |

MD: medical degree.

**Additional Table 3.** Characteristics of respondents to questionnaire 1 (n=66) and questionnaire 2 (n=31)

| **Characteristic** | **Questionnaire 1^a^** | |  | **Questionnaire 2^b^** | |
| --- | --- | --- | --- | --- | --- |
|  | **N** | **Total** |  | **N** | **Total** |
| Age, years, median [IQR] | 64 | 40 [30-53] |  | 31 | 39 [30-52] |
| Females, n (%) | 66 | 35 (53.0) |  | 31 | 16 (51.6) |
| **Main position, n (%)** | 66 |  |  | 31 |  |
| Research |  | 42 (63.6) |  |  | 25 (80.6) |
| Clinical practice |  | 3 (4.5) |  |  | 2 (6.5) |
| Research and clinical practice |  | 21 (31.8) |  |  | 4 (12.9) |
| **Healthcare professionals, n (%)** | 23 |  |  | 6 |  |
| Allied health |  | 13 (56.5) |  |  | 4 (66.7) |
| Nurse |  | 1 (4.3) |  |  | 0 |
| Physician |  | 3 (13.0) |  |  | 0 |
| Surgeon |  | 4 (17.4) |  |  | 1 (16.7) |
| Other |  | 2 (8.7) |  |  | 1 (16.7) |
| **Research experience, years, median [IQR]** | 66 | 11 [4-23] |  | 31 | 15 [4-25] |
| **Highest completed degree, n (%)** | 66 |  |  | 31 |  |
| Bachelor’s degree |  | 3 (4.5) |  |  | 0 |
| Master’s degree |  | 25 (37.9) |  |  | 10 (32.3) |
| Professional degree |  | 2 (3.0) |  |  | 1 (3.2) |
| Doctorate degree |  | 36 (54.5) |  |  | 20 (64.5) |

IQR: interquartile range. Note. ^a^Questionnaire 1: Exploring the agreement on the definition of mobility, conceptual framework, constructs and measures of mobility within physical domain. ^b^Questionnaire 2: Defining, classifying and identifying constructs within physical domain.

**Additional Table 4.** Themes identified in the definition of mobility responses (n=66), assessed in questionnaire 1

| **Theme** | **Frequency (%)**  **(n=66)** |
| --- | --- |
| Move/movement | 50 (75.8) |
| Ability to move | 34 (51.5) |
| Walk | 11 (16.7) |
| Body | 10 (15.2) |
| A to B | 6 (9.1) |
| Environment | 6 (9.1) |
| Transfer | 6 (9.1) |
| With or without assistance/help | 6 (9.1) |
| Independent | 5 (7.6) |
| Range of motion joint | 5 (7.6) |
| Activities | 4 (6.1) |
| Daily | 4 (6.1) |
| ICF definition | 4 (6.1) |
| Move freely/freedom | 4 (6.1) |
| Physical activity | 4 (6.1) |
| Transport | 4 (6.1) |
| Bed | 3 (4.5) |
| Move easily | 3 (4.5) |
| Safe | 3 (4.5) |
| Ambulation | 2 (3.0) |
| Other | 3 (4.5) |

ICF: International Classification of Functioning, Disability and Health.

**Additional Table 5.** Agreement on statements related to the definition of mobility, conceptual framework, constructs and measures of mobility (questionnaire 1) and defining constructs (questionnaire 2)

| **Statement** | **N** | **Strongly agree** | **Agree** | **Neither agree nor disagree** | **Disagree** | **Strongly disagree** | **No opinion** |
| --- | --- | --- | --- | --- | --- | --- | --- |
| **Questionnaire 1** |  |  |  |  |  |  |  |
| ***Definition of mobility*** |  |  |  |  |  |  |  |
| 01. Mobility is clearly defined. | 65 | 0 | 6 (9.2) | 21 (32.3) | 33 (50.8) | 5 (7.7) | N/A |
| 02. Mobility is broadly defined as the ability to move oneself. | 65 | 8 (12.3) | 45 (70.8) | 7 (10.8) | 4 (6.2) | 0 | N/A |
| 03. Mobility includes the context of travel/commuting. | 65 | 4 (6.2) | 44 (67.7) | 10 (15.4) | 6 (9.2) | 1 (1.5) | N/A |
| 04. Mobility includes the use of assistive devices (e.g. wheelchair). | 65 | 13 (20.0) | 45 (69.2) | 3 (4.6) | 4 (6.2) | 0 | N/A |
| 05. Mobility in standardized settings (e.g. lab or clinical environment) requires a different definition than mobility in a daily life environment. | 63 | 3 (4.8) | 25 (39.7) | 16 (25.4) | 14 (22.2) | 5 (7.9) | N/A |
| ***Conceptual framework*** |  |  |  |  |  |  |  |
| 06. A clear conceptual framework defining constructs and measures is required to determine mobility. | 64 | 12 (18.8) | 42 (65.6) | 9 (14.1) | 1 (1.6) | 0 | N/A |
| 07. For health professionals, the WHO ICF is a suitable framework to determine mobility. | 63 | 3 (4.8) | 19 (30.2) | 15 (23.8) | 7 (11.1) | 0 | 19 (30.2) |
| 08. For researchers, the WHO ICF is a suitable framework to determine mobility. | 64 | 3 (4.7) | 17 (26.6) | 11 (17.2) | 16 (25.0) | 1 (1.6) | 16 (25.0) |
| ***Constructs of mobility*** |  |  |  |  |  |  |  |
| 09. Mobility is determined by multiple constructs requiring different measurements. | 64 | 20 (31.3) | 36 (56.3) | 7 (10.9) | 1 (1.6) | 0 | N/A |
| 10. Constructs and measurements to determine mobility are well-defined. | 64 | 0 | 5 (7.8) | 25 (39.1) | 30 (46.9) | 4 (6.3) | N/A |
| 11. Physical activity is an important construct of mobility. | 64 | 11 (17.2) | 32 (50.0) | 8 (12.5) | 12 (18.8) | 1 (1.6) | N/A |
| 12. Muscle status is an important construct of mobility. | 64 | 5 (7.8) | 35 (54.7) | 9 (14.1) | 13 (20.3) | 2 (3.1) | N/A |
| 13. Physical capacity and physical performance are different constructs. | 64 | 25 (39.1) | 33 (51.6) | 4 (6.3) | 2 (3.1) | 0 | N/A |
| 14. The 'can do' and 'do' (i.e. what a person can do and what a person actually does in their daily life) are separate constructs of mobility. | 64 | 19 (29.7) | 37 (57.8) | 3 (4.7) | 5 (7.8) | 0 | N/A |
| 15. Physical performance is defined by what a person actually does in his or her environment. | 64 | 12 (18.8) | 35 (54.7) | 2 (3.1) | 15 (23.4) | 0 | N/A |
| 16. Physical capacity is defined by what an individual can do in a standardised environment. | 64 | 8 (12.5) | 35 (54.7) | 8 (12.5) | 12 (18.8) | 1 (1.6) | N/A |
| ***Mobility measures*** |  |  |  |  |  |  |  |
| 17. Determining mobility requires a standardised core-set of measures. | 64 | 6 (9.4) | 38 (59.4) | 13 (20.3) | 6 (9.4) | 1 (1.6) | N/A |
| 18. Mobility should be measured differently dependent on whether mobility is considered a determinant or outcome. | 64 | 0 | 21 (32.8) | 24 (37.5) | 17 (26.6) | 2 (3.1) | N/A |
| **Questionnaire 2** |  |  |  |  |  |  |  |
| ***Conceptual framework*** |  |  |  |  |  |  |  |
| 01. To define mobility, ICF offers a useful basic conceptual framework for research. | 31 | 4 (12.9) | 19 (61.3) | 5 (16.1) | 3 (9.7) | 0 | N/A |
| 02. Mobility related constructs can be aligned with individual ICF components within the components of body functions and structures, activities and participation, environmental factors, and personal factors. | 31 | 3 (9.7) | 23 (74.2) | 3 (9.7) | 0 | 2 (6.5) | N/A |
| ***Capacity and performance*** |  |  |  |  |  |  |  |
| 03. A construct should be classified under capacity or performance within the ICF depending on how it is measured. | 29 | 4 (13.8) | 19 (65.5) | 3 (10.3) | 2 (6.9) | 1 (3.4) | N/A |
| 04. Capacity is defined by measurements under standardized/ideal conditions. | 30 | 6 (20.0) | 17 (56.7) | 2 (6.7) | 3 (10.0) | 2 (6.7) | N/A |
| 05. Capacity is representing the 'can do,' i.e. what a person is maximally capable of. | 31 | 12 (38.7) | 18 (58.1) | 1 (3.2) | 0 | 0 | N/A |
| 06. Performance is defined by measurements embedded within a (daily) task/activity. | 30 | 6 (20.0) | 20 (66.7) | 1 (3.3) | 2 (6.7) | 1 (3.3) | N/A |
| 07. Performance is representing the 'do' i.e. what a person actually does in their daily life. | 29 | 6 (20.7) | 17 (58.6) | 2 (10.3) | 3 (10.3) | 0 | N/A |
| 08. The term physical performance is often used instead of physical capacity; this terminology should be used appropriately to avoid confusion. | 29 | 13 (44.8) | 14 (48.3) | 2 (6.9) | 0 | 0 | N/A |
| ***Function*** |  |  |  |  |  |  |  |
| 09. The term function refers to body functions. | 30 | 5 (16.7) | 12 (40.0) | 7 (23.3) | 5 (16.7) | 1 (3.3) | N/A |
| 10. The term function means the same as the term capacity. | 30 | 0 | 1 (3.3) | 3 (10.0) | 14 (46.7) | 12 (40.0) | N/A |
| 11. The terms function and functioning have different definitions. | 29 | 3 (10.3) | 19 (65.5) | 2 (6.9) | 4 (13.8) | 1 (3.4) | N/A |
| 12. Physical function is an umbrella term for lower extremity function, upper extremity function, back and neck function, and (instrumental) ADL. | 30 | 1 (3.3) | 13 (43.3) | 6 (20.0) | 10 (33.3) | 0 | N/A |
| 13. Muscle function is an umbrella term for muscle strength, muscle power and muscle endurance. | 30 | 7 (23.3) | 21 (70.0) | 0 | 2 (6.7) | 0 | N/A |
| 14. Muscle function can also be termed muscle capacity. | 30 | 4 (13.3) | 12 (40.0) | 2 (6.7) | 10 (33.3) | 2 (6.7) | N/A |
| 15. Muscle function can also be termed muscle quality. | 30 | 0 | 7 (23.3) | 4 (13.3) | 17 (56.7) | 2 (6.7) | N/A |

Data are presented as n (%). WHO: World Health Organization, ICF: International Classification of Functioning, Disability and Health, ADL: activities of daily living.

**Additional Table 6.** Reported constructs of mobility, assessed in questionnaire 1

| **Construct** | **Freq. (%)** | **Construct** | **Freq. (%)** |
| --- | --- | --- | --- |
| Physical activity | 10 (6.37) | Gait quality | 1 (0.64) |
| Assistive devices | 8 (5.10) | Getting to places | 1 (0.64) |
| ADL | 6 (3.82) | Goal-directness | 1 (0.64) |
| Ability to move | 5 (3.18) | How often/the intensity that a person is moving from one place to another | 1 (0.64) |
| Balance | 5 (3.18) | Intensity | 1 (0.64) |
| Walking/ability to walk | 5 (3.18) | Lifestyle | 1 (0.64) |
| Capacity | 4 (2.55) | Limitations | 1 (0.64) |
| Joint (function, mobility, range of motion) | 4 (2.55) | Meaningful for the patient | 1 (0.64) |
| Movement | 4 (2.55) | Mobilize | 1 (0.64) |
| Performance | 4 (2.55) | Motor control | 1 (0.64) |
| Physical function | 4 (2.55) | Muscle mass | 1 (0.64) |
| Health (status) | 3 (1.91) | Oxidative capacity | 1 (0.64) |
| Muscle strength | 3 (1.91) | Participation in leisure activities | 1 (0.64) |
| Physical capacity | 3 (1.91) | Participation in society activities | 1 (0.64) |
| Climbing stairs | 2 (1.27) | Participation in sports | 1 (0.64) |
| Cognition | 2 (1.27) | Participation in the community | 1 (0.64) |
| Environment | 2 (1.27) | Perform physically in a continuous manner allowing an individual to move sufficient or towards their goal | 1 (0.64) |
| Exercise capacity | 2 (1.27) | Personal characteristics | 1 (0.64) |
| Mental health | 2 (1.27) | Physical | 1 (0.64) |
| Motivation | 2 (1.27) | Physical behaviour | 1 (0.64) |
| Muscle function | 2 (1.27) | Physical condition | 1 (0.64) |
| Participation | 2 (1.27) | Physical health | 1 (0.64) |
| Quality of movement | 2 (1.27) | Proprioception | 1 (0.64) |
| Range of motion | 2 (1.27) | Quality of the environment | 1 (0.64) |
| Transportation | 2 (1.27) | Quantity | 1 (0.64) |
| Abilities | 1 (0.64) | Quantity of movement | 1 (0.64) |
| Ability to participate | 1 (0.64) | Range in which movement is possible | 1 (0.64) |
| Accessibility | 1 (0.64) | Required artificial tools | 1 (0.64) |
| Aerobic capacity | 1 (0.64) | Required physical performance | 1 (0.64) |
| Anthropometry | 1 (0.64) | Running | 1 (0.64) |
| Arm use | 1 (0.64) | Self-perceived competence | 1 (0.64) |
| Arthrosis | 1 (0.64) | Self-care | 1 (0.64) |
| Biking | 1 (0.64) | Sensation | 1 (0.64) |
| Body | 1 (0.64) | Societal limitations | 1 (0.64) |
| Boundaries | 1 (0.64) | Stability | 1 (0.64) |
| Change in mobility | 1 (0.64) | The way you move from A to B | 1 (0.64) |
|  |  |  |  |
| Coordination | 1 (0.64) | To what degree (regarding speed, distance, endurance) can a patient/participant move around within their own environment or in an institution | 1 (0.64) |
| Efficiency | 1 (0.64) | Transfer | 1 (0.64) |
| Effort of movements | 1 (0.64) | Transferring from one position to another | 1 (0.64) |
| Emotional limitations | 1 (0.64) | Travelling | 1 (0.64) |
| Energy | 1 (0.64) | Travelling distance | 1 (0.64) |
| Extent to which a physical body or body parts can actively move and function | 1 (0.64) | Types of activities that can be performed | 1 (0.64) |
| Falls | 1 (0.64) | Walking distance | 1 (0.64) |
| Freedom and independence | 1 (0.64) | Walking performance | 1 (0.64) |
| Functional capacity to move | 1 (0.64) | Weight-bearing activity | 1 (0.64) |
| Functional context | 1 (0.64) | Willingness | 1 (0.64) |

Freq.: frequency. ADL: Activities of daily living.

**Additional Table 7.** Reported measures of mobility, assessed in questionnaire 1

| **Measure** | **Freq. (%)** | **Measure** | **Freq. (%)** |
| --- | --- | --- | --- |
| Physical activity | 19 (11.1) | Joint mobility examinations | 1 (0.58) |
| Walking test | 18 (10.5) | Joint range of motion | 1 (0.58) |
| Muscle strength | 9 (5.23) | Jumping power | 1 (0.58) |
| SPPB | 8 (4.65) | Level of community walking | 1 (0.58) |
| ADL | 7 (4.07) | Maximal oxidative capacity | 1 (0.58) |
| Balance | 6 (3.49) | Mobility hip/ knee/ spine | 1 (0.58) |
| Gait quality | 4 (2.33) | Mobility of joints | 1 (0.58) |
| Aids | 3 (1.74) | Mobility questionnaire | 1 (0.58) |
| AM-PAC | 3 (1.74) | Mobility scores (JH-HLM) | 1 (0.58) |
| Chair stand | 3 (1.74) | Modified Iowa Level of Assistance Scale | 1 (0.58) |
| DEMMI | 3 (1.74) | Motion capture (e.g. joint angles) | 1 (0.58) |
| Range of motion | 3 (1.74) | Motricity index | 1 (0.58) |
| Step test | 3 (1.74) | Muscle activity | 1 (0.58) |
| Adaptability of gait | 2 (1.16) | Muscle extensibility | 1 (0.58) |
| Capacity | 2 (1.16) | Muscle length | 1 (0.58) |
| Muscle function | 2 (1.16) | Muscle mass | 1 (0.58) |
| Performance | 2 (1.16) | O2 energy use | 1 (0.58) |
| Physical functioning | 2 (1.16) | Oxidative capacity | 1 (0.58) |
| Physical performance | 2 (1.16) | Participatory factors | 1 (0.58) |
| TUG | 2 (1.16) | Patient reported physical functioning | 1 (0.58) |
| Abilities | 1 (0.58) | Patient survey | 1 (0.58) |
| Abilities/disabilities | 1 (0.58) | Patio-temporal features | 1 (0.58) |
| Ability to walk | 1 (0.58) | Performance of work-related activities as outcome | 1 (0.58) |
| Aerobic capacity | 1 (0.58) | Physical restrictions during daily life | 1 (0.58) |
| AMEXO | 1 (0.58) | PROMIS PF | 1 (0.58) |
| Bedridden | 1 (0.58) | Proms (physical functioning) | 1 (0.58) |
| Behavioural outcomes | 1 (0.58) | Quality of daily activity (stand, sit, walk) | 1 (0.58) |
| Body functions outcomes (incl. muscle performance) | 1 (0.58) | Quality of gait/ transfers | 1 (0.58) |
| Contextual factors | 1 (0.58) | Quantity | 1 (0.58) |
| Daily weight-bearing activities (frequency and duration) | 1 (0.58) | Reactive gait performance | 1 (0.58) |
| Degree of arthrosis | 1 (0.58) | Rockwood frailty | 1 (0.58) |
| Determinants of mobility | 1 (0.58) | Sensation tests (Nottingham’s) | 1 (0.58) |
| Dynamics of gas transport during exercise development of lactic acidosis | 1 (0.58) | SF-36 PF | 1 (0.58) |
| Efficiency | 1 (0.58) | Stability | 1 (0.58) |
| Exercise capacity | 1 (0.58) | Tendon extensibility | 1 (0.58) |
| Expanded disability status scale | 1 (0.58) | Tendon length | 1 (0.58) |
| FAC | 1 (0.58) | Time out of bed | 1 (0.58) |
| Fitness | 1 (0.58) | Torque of a joint | 1 (0.58) |
| Functional range of motion | 1 (0.58) | Transportation means | 1 (0.58) |
| Gait analysis | 1 (0.58) | Trunk control test | 1 (0.58) |
| Gait performance | 1 (0.58) | Walking free | 1 (0.58) |
| Gait stability | 1 (0.58) | Wearables | 1 (0.58) |
| Groninger frailty index | 1 (0.58) | Work-related activities as risk factors for an occupational disease | 1 (0.58) |
| Heart/lung capacity | 1 (0.58) | X-ECG/CPET | 1 (0.58) |
| Intensity | 1 (0.58) |  |  |

Freq.: frequency. ADL: Activities of daily living. AM-PAC: Activity Measure for Post-Acute Care. DEMMI: de Morton Mobility Index. FAC: Functional Ambulation Classification. JH-HLM: John Hopkins Highest Level of Mobility. PROMIS PF - Patient-Reported Outcomes Measurement Information System Physical Function. SF-36 PF - Short Form-36 physical functioning. SPPB: Short Physical Performance Battery. TUG: Timed Up and Go. X-ECG: exercise electrocardiogram. CPET: Cardiopulmonary Exercise Testing.

**Additional Table 8.** Assessment of classification of constructs using the ICF components, assessed in questionnaire 2

| **Question** |  | **Classification** | | | | | |
| --- | --- | --- | --- | --- | --- | --- | --- |
| **[…] should be classified under:** | **N** | **Body functions and structures** | **Activities and participation - Capacity** | **Activities and participation - Performance** | **Activities and participation - Capacity & Performance** | **All of the above, depending on what and how it is measured** | **Unsure** |
| Functions | 30 | 20 (66.7) | 2 (6.7) | 1 (3.3) | N/A | 5 (16.7) | 2 (6.7) |
| Functioning | 30 | 2 (6.7) | 0 | 14 (46.7) | N/A | 13 (43.3) | 1 (3.3) |
| Muscle function | 30 | 25 (83.3) | 2 (6.7) | 0 | 3 (10.0) | N/A | 0 |
| Muscle quality | 30 | 22 (73.3) | 2 (6.7) | 0 | 2 (6.7) | N/A | 4 (13.3) |
| Quality of movement | 29 | 3 (10.3) | 1 (3.4) | 9 (31.0) | 11 (37.9) | N/A | 5 (17.2) |
| Gait function | 30 | 7 (23.3) | 7 (23.3) | 5 (16.7) | 7 (23.3) | N/A | 4 (13.3) |
| Balance | 30 | 9 (30.0) | 7 (23.3) | 7 (23.3) | 6 (20.0) | N/A | 1 (3.3) |
| (Instrumental) ADL | 30 | 1 (3.3) | 2 (6.7) | 17 (56.7) | 9 (29.0) | N/A | 1 (3.3) |
| Exercise capacity | 30 | 8 (26.7) | 14 (46.7) | 2 (6.7) | 5 (16.7) | N/A | 1 (3.3) |
| Oxidative capacity | 30 | 16 (53.3) | 9 (30.0) | 0 | 2 (6.7) | N/A | 3 (10.0) |
| Exercise endurance | 30 | 10 (33.3) | 13 (43.3) | 2 (6.7) | 4 (13.3) | N/A | 1 (3.3) |
|  | **N** | **Another word for mobility with the same definition** | **A construct within: body functions and structures** | **A construct within: activities and participation - capacity** | **A construct within: activities and participation - performance** | **A separate construct within activities and participation, next to capacity and performance** | **Unsure** |
| Physical function is: | 30 | 1 (3.3) | 14 (46.7) | 1 (3.3) | 5 (16.7) | 3 (10.0) | 6 (20.0) |
| Human movement is: | 30 | 5 (16.7) | 5 (16.7) | 0 | 5 (16.7) | 6 (20.0) | 9 (30.0) |
| Ambulation is: | 28 | 8 (28.6) | 2 (7.1) | 2 (7.1) | 8 (28.6) | 4 (14.3) | 4 (14.3) |

Data is presented as n (%). ICF: International Classification of Functioning, Disability and Health. ADL: activities of daily living.
